# Meta-analysis of tumor necrosis factor alpha -308 polymorphism and knee osteoarthritis risk

**DOI:** 10.1186/1471-2474-15-373

**Published:** 2014-11-15

**Authors:** Suotang Kou, Yaochi Wu

**Affiliations:** Acupuncture Moxibustion and Tuina traumatology of Shanghai Jiao Tong University Affiliated Sixth People’s Hospital, Shanghai, China

**Keywords:** TNF-α, -308 polymorphism, Osteoarthritis, Meta-analysis

## Abstract

**Background:**

Several case–control studies have been conducted to clarify the association between the tumor necrosis factor alpha (TNF-α) -G308A polymorphism and risk of osteoarthritis (OA); however, the results are inconsistent. This meta-analysis was performed to clarify this issue using all the available evidence.

**Methods:**

Eligible articles were retrieved by searching PubMed, Web of science and Google scholar. The strength of the association between the TNF-α -G308A polymorphism and risk of OA was assessed by odds ratios (ORs) with the corresponding 95% confidence interval (CI) for each study.

**Results:**

Seven studies were included in the meta-analysis, which included 983 OA cases and 1355 controls. The pooled analysis based on all included studies showed a significantly increased OA risk in the recessive genetic model analysis (OR = 11.08, 95% CI = 4.75-25.86, p < 0.001) and in the A allele *vs*. G allele analysis (OR = 2.30, 95% CI = 1.08-4.90). However, there was no statistical difference in the dominant genetic model analysis (OR = 2.45, 95% CI = 0.95-6.27, p = 0.06). Furthermore, we found that OA patients had a higher frequency of the AA genotype (OR = 10.49, 95% CI = 4.47-24.61) and GA genotype (OR = 1.78, 95% CI = 1.03-3.08) compared with the control population.

**Conclusion:**

Our results suggested that the TNF-α -G308A polymorphism were associated with an increased risk of OA.

**Electronic supplementary material:**

The online version of this article (doi:10.1186/1471-2474-15-373) contains supplementary material, which is available to authorized users.

## Background

As one of the most common age-related disease leading to restrictions in daily activities of the elderly, osteoarthritis (OA) is set to become the 4th-highest impact condition in women and the 8th-highest in men. The prevalence of OA is steadily increasing across all age groups [1]. Given the high morbidity and huge economic burden of OA, there is an urgent need to define the pathogenic factors involved in OA development. OA is a multistep, multifactorial disease that involves a complex interplay between genetic and environmental factors. Besides the traditional common risk factors, such as aging, obesity, previous injury, smoking habit and hormone therapy [2], recent studies have revealed that inflammatory processes play a pivotal role in OA pathogenesis [3]. Proinflammatory cytokines are now implicated as important mediators in OA, and TNF-α and interleukin 1 beta (IL-1β) are considered the major factors.

TNF-α is a multifunctional pro-inflammatory cytokine involved in various physiological and pathological processes, including inflammation, immunoregulation, proliferation and apoptosis [4]. TNF-α is produced by chondrocytes, mononuclear cells, osteoblasts and synovial tissues, and can stimulate its own production and induce chondrocytes and synovial cells to produce other cytokines. TNF-α also induces osteoclastic bone resorption [5] and destruction of cartilages [6]. Elevated levels of TNF-α have been found in the synovial fluid, synovial membrane, subchondral bone and cartilage of OA patients [7, 8], confirming its important roles in OA pathogenesis.

Genetic variations within the TNF-α promoter could alter the transcription and translation of TNF-α [9]. Polymorphisms in the promoter region of TNF-α have been found at -G238A, -G308A, -C863A, and -C857A, among which, the -G308A polymorphism is the most commonly studied. Previous studies found that the expression of TNF-α is higher in patients carrying the -308A allele than in those carrying the -308G allele, suggesting that this polymorphism has functional implications in inflammation and may be associated with inflammatory diseases [10]. In fact, previous studies have demonstrated the association of this polymorphism with certain inflammatory diseases, such as fatty liver disease [11], hepatitis B virus infection [12], systemic lupus erythematosus [13], inflammatory bowel disease [14], arteritis [15] and rheumatoid arthritis [16]. Several case–control studies have been conducted to clarify the association between the TNF-α -G308A polymorphism and risk of OA [17–23]; however, the results are inconsistent. Han’s study [20] and Ji’s study [22] found that the frequency of A allele was significantly higher in the OA cases; however, Sezgin’s study [19] and Valle’s study [21] found no association between the TNF-α -G308A polymorphism and OA risk. In view of the uncertain association between TNF-α -G308A polymorphism and OA risk, we sought to obtain more precise information by conducting a meta-analysis including all of the evidence produced to date.

## Methods

### Search strategy

Eligible articles were retrieved by searching the PubMed bibliographical database, Web of science and Google scholar (up to January 16, 2014) using the following combination of keywords: (tumour necrosis factor-alpha) OR (tumour necrosis factor-α) OR (TNF-α)); OA OR osteoarthritis; polymorphism OR polymorphisms OR variants OR variant. In addition, we checked the references in reviews and in the retrieved articles to avoid missing any relevant studies. There was no restriction on language in the search. The article selection was carried out by both YW and SK independently to minimize bias and human error.

### Inclusion and exclusion criteria

To be included in this meta-analysis, an article had to provide the following information: 1) the number of OA cases and controls; and 2) the number of individuals with GG, GA and AA in both OA cases and controls. Those not designed as case–control studies, systemic reviews, and those that provided no usable data were excluded.

### Data extraction

Two independent reviewers used a predesigned data extraction table to extract the data. The following information was extracted from each included article: journal name, first author, publication year, ethnicity and country, OA phenotype, source of controls, the number of genotypes in OA cases and controls, and the results of the study. Disagreement was resolved by discussion.

### Statistical analysis

The strength of the association between the TNF-α -G308A polymorphism and risk of OA was assessed by odds ratios (ORs) with the corresponding 95% confidence interval (CI) for each study. The ORs and their 95% CIs were assessed for the following genetic models: 1) the dominant genetic model (AA + GA *vs*. GG) (A stands for the minor allele and G stands for the major allele); 2) the recessive genetic model (AA *vs*. GA + GG) and 3) the allele A *vs*. allele G analysis. The genotype frequencies of GG, GA and, AA were also assessed using the same method. A chi-squared (χ2) test was used to assess heterogeneity across studies. A fixed effect model was used when no heterogeneity existed among the studies. Otherwise, a random effect model was used. Meta-regression analysis was performed to find any sources of heterogeneity. Subgroup analysis for ethnicity (Asian and Caucasian), design type (HCC (hospital based case–control study) and PCC (population based case–control study)) and sample size (smaller (total sample <300) and larger (total sample ≥300)) was conducted. Potential publication bias was assessed using a funnel plot, and calculated using Begg’s and Egger’s tests (P < 0.05 was considered as significant publication bias). Influence analysis was performed by omitting each study to find potential outliers. In the control populations, the Hardy-Weinberg equilibrium (HWE) was tested using a chi- squared (χ2) test. Two authors performed the statistical analysis independently and obtained the same results. STATA software (version 11; Stata Corporation, College Station, TX, USA) was used to perform the statistical analyses. P values less than 0.05 were considered statistically significant.

## Results

### Literature selection and study characteristics

**Figure 1:**
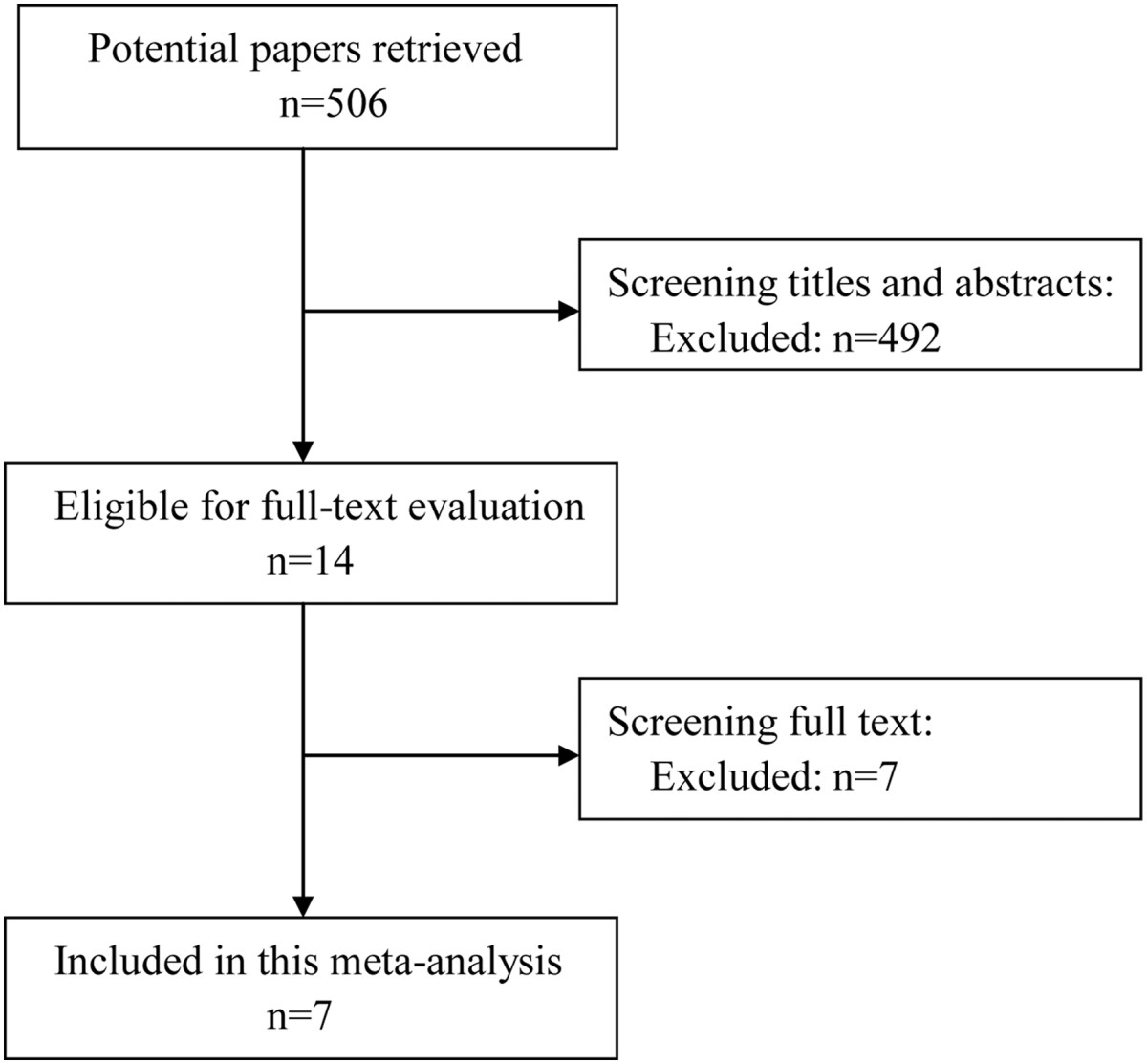
**Flowchart of the study selection.**

A total of 506 articles were retrieved from PubMed, Web of science and Google Scholar, 492 of which were excluded after screening the titles and abstracts (469 were irrelevant studies and 23 were duplicate studies). Fourteen articles were selected for detailed assessment and seven of them were excluded (three were not about G308A, three were not about OA risk and one had no usable data). Finally, seven studies were included in this meta-analysis, which included 983 OA cases and 1355 controls. The detailed selection procedure is shown in Figure 1. Genotype distributions in the controls of Moos’s study [17] were not in agreement with HWE. Most of the cases were knee OA, only one study recruited some hip OA cases [17]. The detailed characteristics of the included studies are shown in Table 1.

**Table 1:** **Characteristics of studies included in TNF-α polymorphisms and osteoarthritis**

Study	Ethnicity	Design	Phenotype	HWE	Total cases	Total controls	GG		GA		AA	
							Cases	Controls	Cases	Controls	Cases	Controls
Moos 2000 [17]	Caucasian	HCC	Knee/Hip	No	55	240	36	166	18	74	1	0
Romero 2002 [18]	Caucasian	HCC	Knee	Yes	31	30	28	28	3	2	0	0
Sezgin 2008 [19]	Caucasian	HCC	Knee	Yes	151	84	121	72	26	12	4	0
Han 2012 [20]	Asian	PCC	Knee	Yes	301	291	79	258	188	33	34	0
Valle 2012 [21]	Caucasian	PCC	Knee	Yes	50	100	44	93	6	7	0	0
Ji 2013 [22]	Asian	PCC	Knee	Yes	200	305	143	253	50	50	7	2
Cheng 2013 [23]	Asian	PCC	Knee	Yes	200	305	143	253	50	49	7	3

*HCC* Hospital based case–control study, *PCC* population based case–control study. *HWE* Hardy-Weinberg equilibrium.

### Quantitative data synthesis

**Figure 2:**
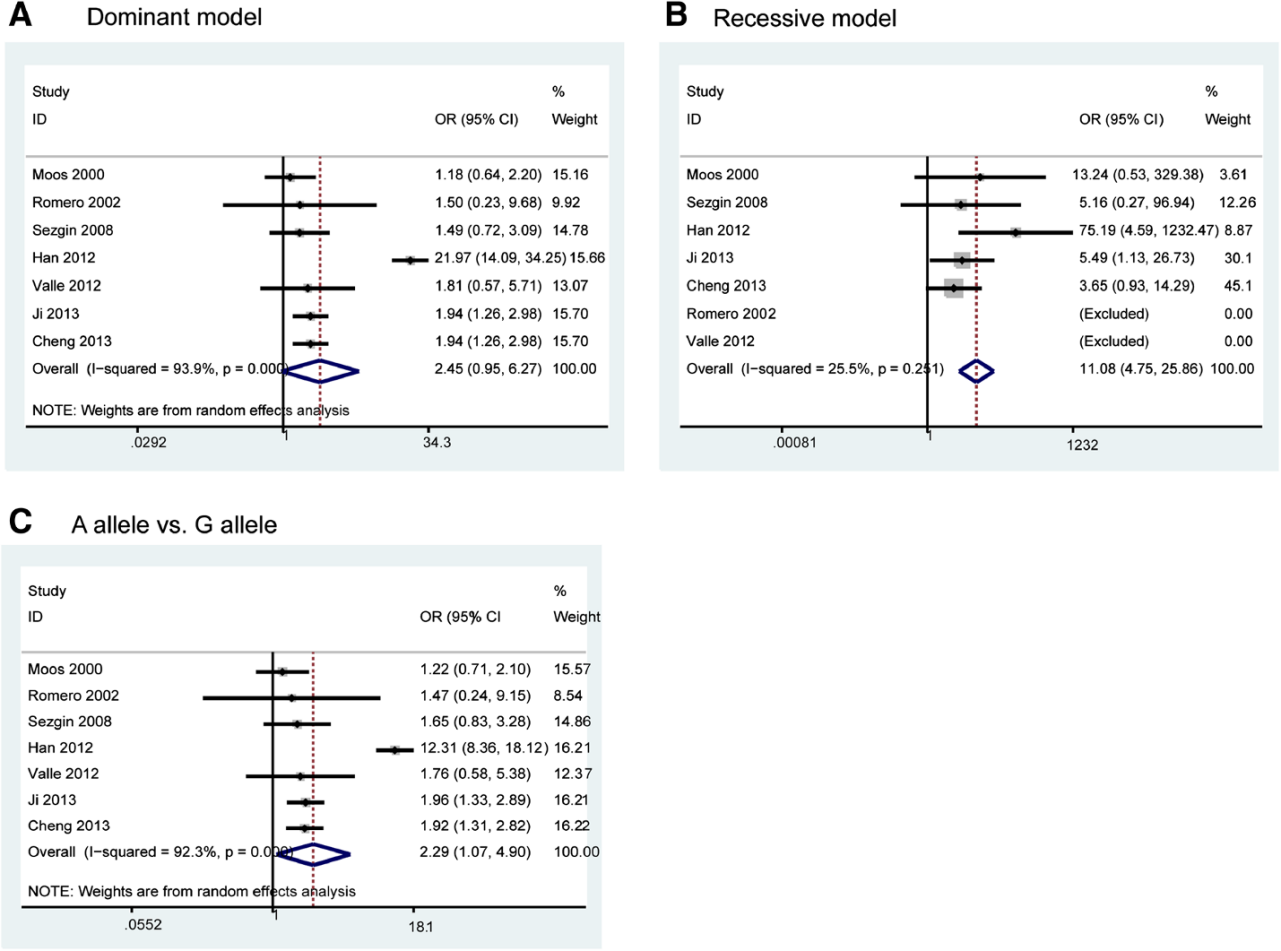
**Meta-analysis of TNF-α -308 polymorphism and OA risk: (A) dominant genetic model analysis; (B) recessive genetic model analysis (C) A allele vs. G allele analysis.** Note: No AA genotype in Romero’s study and Valle’s study, therefore, these two studies were excluded in the recessive model analysis.

**Figure 3:**
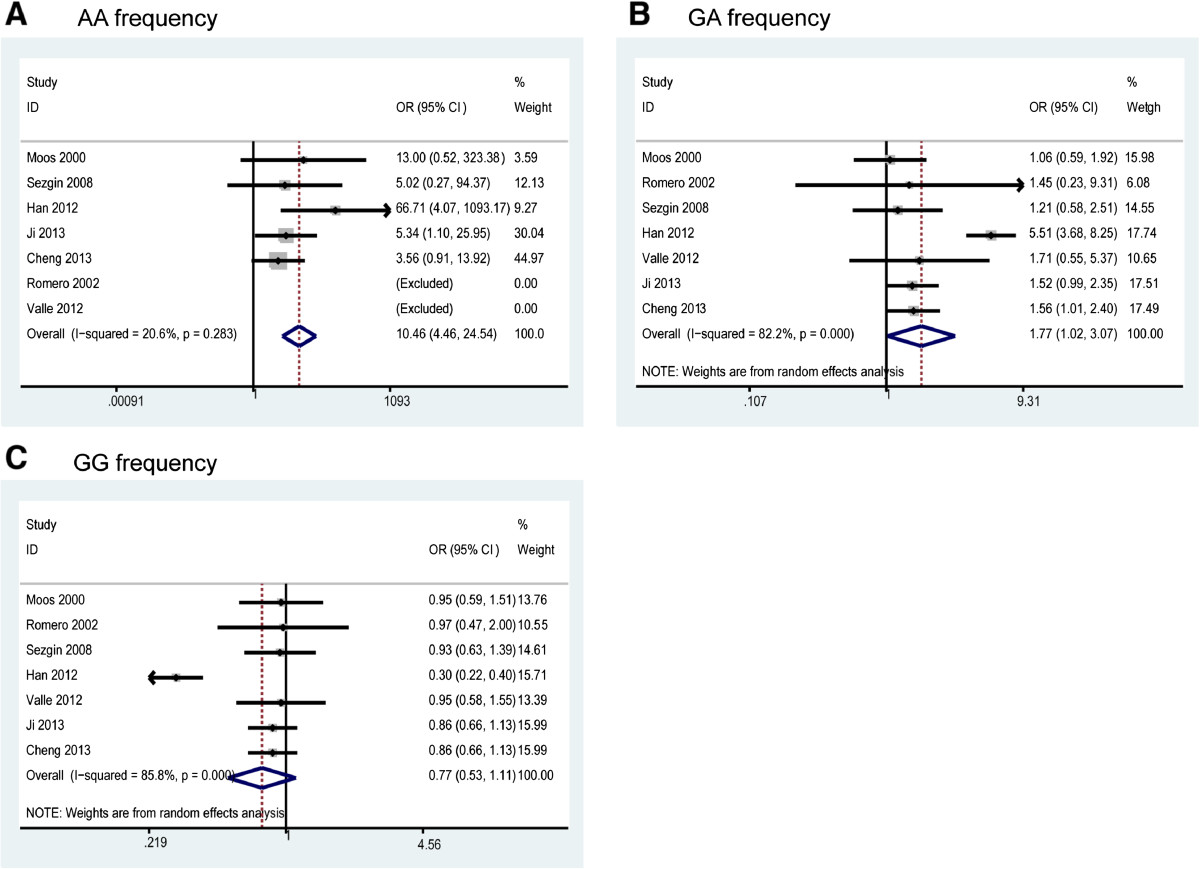
**Meta-analysis of TNF-α -308 genotypes and OA risk: (A) AA genotype frequency; (B) GA genotype frequency; (C) GG genotype frequency.** Note: No AA genotype in Romero’s study and Valle’s study, therefore, these two studies were excluded in AA frequency analysis.

The pooled analysis based on all included studies showed a significantly increased risk in the recessive genetic model analysis (OR = 11.08, 95% CI = 4.75-25.86, p < 0.001) (Figure 2B) and in the A allele *vs*. G allele analysis (OR = 2.30, 95% CI = 1.08-4.90, p < 0.001) (Figure 2C). However, there was statistically non-significant tendency towards decreased risk in the dominant genetic model analysis (OR = 2.45, 95% CI = 0.95-6.27, p = 0.06) (Figure 2A).We then assessed the genotype frequencies based on all included studies. The results showed that OA patients had a higher frequency of the AA genotype (OR = 10.49, 95% CI = 4.47-24.61, p = 0.001) (Figure 3A) and GA genotype (OR = 1.78, 95% CI = 1.03 -3.08, p = 0.006) (Figure 3B) compared with the control population. However, there was no significant difference for the GG genotype (OR = 0.77, 95% CI = 0.53-1.12, p = 0.14) (Figure 3C). The detailed results are shown in Table 2.

**Table 2:** **Summary ORs and 95**% **CIs of TNF-α polymorphism and osteoarthritis**

Analysis	n	Dominant model(GA + AA vs.GG)		Recessive model(AA vs.GG + GA)		A allele vs. G allele	
		OR(95% CI)	P/P_het_	OR(95% CI)	P/P_het_	OR(95% CI)	P/P_het_
**Overall**	7	2.45(0.95-6.27)	0.06/0.00	11.08(4.75-25.86)	0.00/0.25	2.30(1.08-4.90)	0.03/0.00
**Ethnicity**							
Caucasian	2	1.37(0.89-2.10)	0.14/0.92	6.99(0.70-69.68)	0.10/0.66	1.42(0.96-2.09)	0.08/0.89
Asian	3	4.35(0.91-20.92)	0.07/0.00	11.85(4.77-29.48)	0.00/0.07	3.59(1.06-12.18)	0.04/0.00
**Study design**							
HCC	2	1.31(0.83-2.08)	0.24/0.89	6.99(0.70-69.68)	0.10/0.66	1.38(0.91-2.08)	0.13/0.79
PCC	3	3.58(0.94-13.62)	0.06/0.00	11.85(4.77-29.48)	0.00/0.07	3.10(1.08-8.87)	0.04/0.00
**Sample size**							
Smaller	2	1.37(0.89-2.10)	0.14/0.92	6.99(0.70-69.68)	0.10/0.66	1.42(0.96-2.09)	0.08/0.89
Larger	3	4.35(0.91-20.92)	0.07/0.00	11.85(4.77-29.48)	0.00/0.07	3.59(1.06-12.18)	0.04/0.00

n, number of studies in each analysis; HCC, Hospital based case–control study; PCC, population based case–control study; OR, odds ratio; CI, confi dence interval; p, p value for each test; Phet, p -value for heterogeneity test.

### Tests of heterogeneity and subgroup analysis

We found heterogeneities in the dominant genetic model analysis (p < 0.01). A random effects model was adopted in these two analyses. Furthermore, a meta-regression analysis was conducted to find potential sources of heterogeneities. Unfortunately, the common variables, such as publication year, ethnicity, study design type and sample size were not significant sources of heterogeneities. However, we still performed subgroup analysis based on ethnicity, study design type (HCC or PCC,) and total sample size, because such subgroup analysis was valuable. The detailed results are shown in Table 2.

### Sensitivity analysis

**Figure 4:**
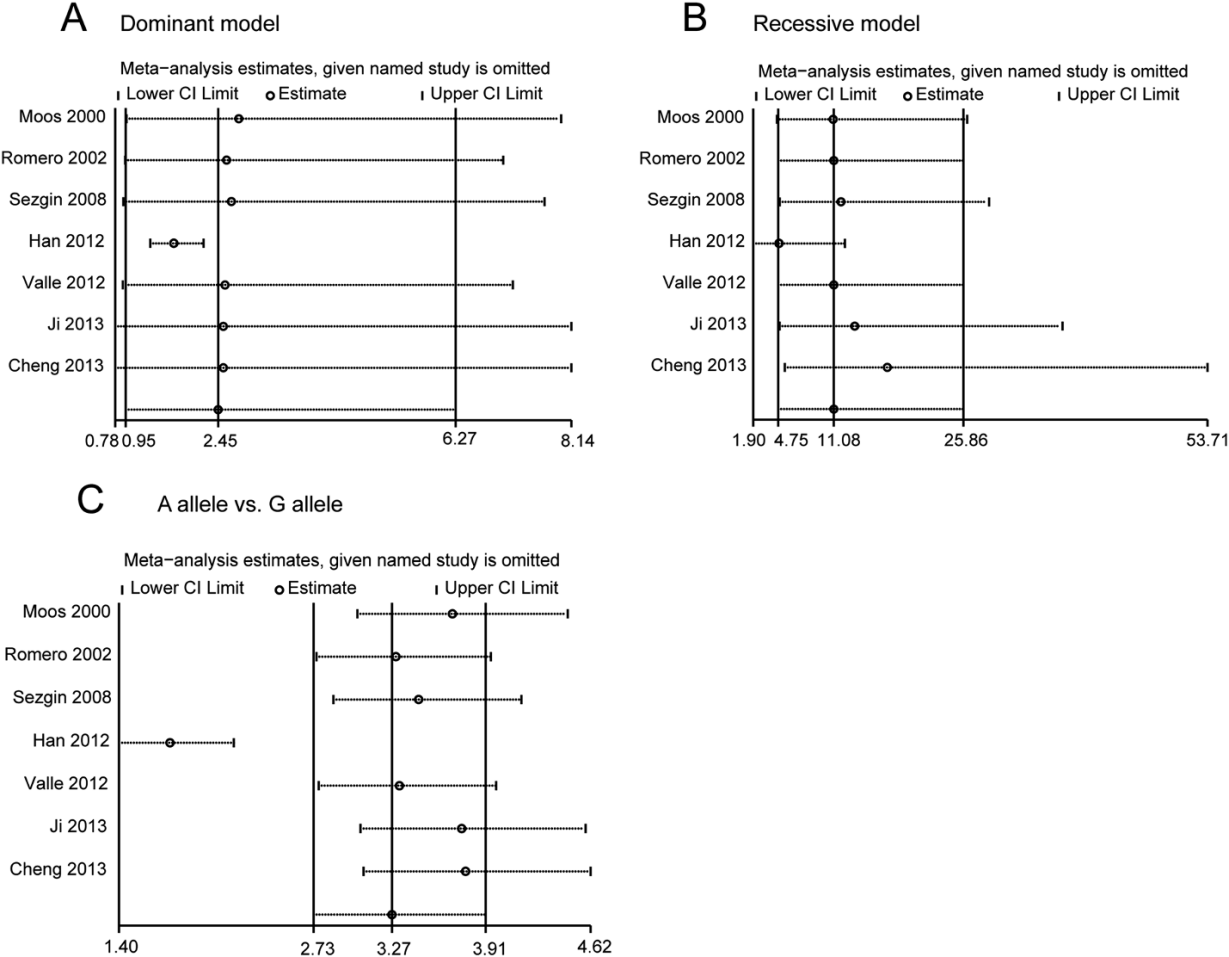
**Influence analysis for TNF-α -308 polymorphism in the overall analysis: (A) dominant model analysis; (B) recessive model analysis; (C) A allele vs. G allele analysis.** These figures show the influence of individual studies on the pooled ORs. The middle vertical axis represents the overall ORs and the two vertical axes represent its 95% CIs. Open circles represent the pooled OR when the selected study is omitted in this meta-analysis. The two ends of the dotted lines indicate the 95% CI.

Influence analysis was performed to assess the sensitivity of each individual study on the pooled ORs, by sequential omission of each individual study. The results suggested that Han’s study [20] significantly affected almost all of the pooled ORs (Figure 4A-C). Therefore, we calculated the results again with Han’s study [20] omitted. The results are shown in Table 3 and Additional file 1: Figure S1; Additional file 2: Figure S2. The pooled analysis showed an increased risk in all three analyses: the dominant model analysis (OR = 1.72, 95% CI = 1.35 -2.20, p < 0.001), the recessive model analysis (OR = 4.84, 95% CI = 1.90 -12.35, p = 0.001) and A allele vs. G allele analysis (OR = 1.75, 95% CI = 1.40 -2.19, p < 0.001) (Additional file 1: Figure S1A-C). We also re-assessed the genotype frequency of GG, GA and AA; however, the results were almost the same as those including all the studies (Additional file 2: Figure S2A-C).

**Table 3:** **Summary ORs and 95**% **CIs after ommiting Han’s study**

Analysis	OR(95% CI)	p value	P_het_
Dominant model	1.72(1.35-2.20)	<0.001	0.82
Recessive model	4.84(1.90-12.35)	0.001	0.90
A allele vs. G allele	1.75(1.40-2.19)	<0.001	0.8
GG frequency	0.89(0.77-1.04)	0.14	0.99
GA frequency	1.41(1.10-1.80)	0.006	0.91
AA frequency	4.72(1.85-12.03)	0.001	0.90

Dominant model: GA + AA vs. GG; Recessive model, AA vs. GA + GG; p, p value for test; Phet, p value for heterogeneity test.

### Publication bias

**Figure 5:**
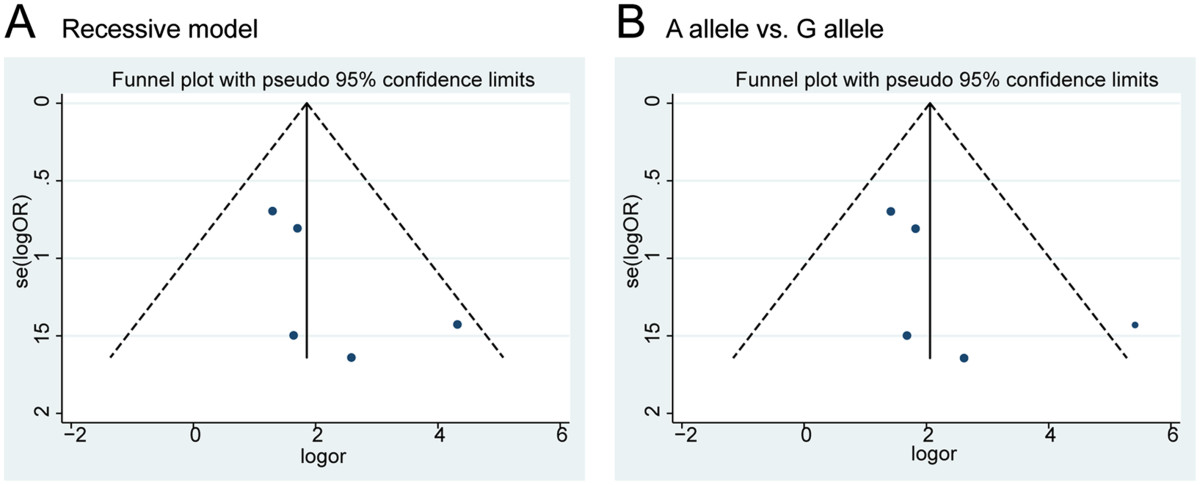
**Funnel plot of TNF-α -308 genotypes and OA risk for publication bias: (A) recessive model analysis; (B) A allele vs. G allele.**

Funnel plots were used to estimate qualitatively the potential publication bias. Taking the the recessive model analysis and A allele vs. G allele analysis as examples, the shapes of the funnel plots did not indicate any obvious asymmetry (Figure 5A,B). Furthermore, Begg’s and Egger’s tests were used to estimate quantitatively the potential publication bias. The p values were all greater than 0.05, indicating no publication bias.

## Discussion

To the best of our knowledge, this is the first meta-analysis to evaluate the association between the TNF-α -308 polymorphism and risk of OA. In this meta-analysis, we discovered an increased OA risk in the recessive genetic model analysis and in the A allele vs. G allele analysis. Furthermore, we found a higher frequency of the AA and GA genotype in OA patients.

TNF-α is a potent pro-inflammatory cytokine in inflammatory diseases and the immune response. Higher levels of TNF-α were found in OA patients compared with the control population [24]. TNF-α can induce the production of other cytokines, matrix metalloproteinases and prostaglandins 9, and inhibit the synthesis of proteoglycans and type II collagen; thus it plays a pivotal role in cartilage matrix degradation and bone resorption in OA [25, 26]. Single nucleotide conversion from guanine (G) to adenine (A) at position -308 is the most common polymorphism in general populations and this transition has been shown to influence the expression of TNF-α. According to previous study, the -308 allele A is associated with about 6 fold increased transcriptional activity and higher protein levels of TNF-α [27]. Valle’s study [21] also found that OA patients with -308 GG genotype expressed less TNF-α mRNA than patients with the GA genotype. Therefore, the TNF-α -308 polymorphism may have some effect on the hosts’ susceptibility to OA by altering TNF-α expression. In support of this, our meta-analysis found an increased risk of OA in the AA *vs*. GG analysis and the recessive model analysis, and higher frequencies of the AA and GA genotypes in OA patients. However, by subgroup analysis, we found that the relationship of TNF-α-308 polymorphism and OA risk only existed among Asian population but not among Caucasion population. In recent GWAS studies, 11 OA susceptibility loci with genome-wide significance levels was indentified. Among them, a region containing HLA class II/III genes, which close to the region habouring TNF gene, showed association in Asians but not in Europeans [28–30], other nine loci reached genome-wide significance in Europeans[31]. These findings exactly explained why the relationship of TNF-α-308 polymorphism and OA risk only existed among Asian population but not among Caucasion population.

Influence analysis suggested that Han’s study [20] might have significantly affected the results; however, when omitting Han’s study, the conclusions showed good consistency with the previous results. Therefore, the conclusions from our meta- analysis are sound and reliable.

Recently, we have noticed that some studies have been conducted to explore the efficacy of anti-TNF therapy on OA. Güler-Yüksel’s study have found that treatment with TNF-α inhibitor might reduce hand OA in patients with rheumatoid arthritis[32], which shed light to the role of anti-TNF-α in treatment of hand OA. However, a recently published RCT failed to confirm the efficacy of anti-TNF-α in treatment of primary hand OA [33], and the author speculated that TNF α is not the right target in order to improve symptoms in hand OA and TNF α blockers may exert a differential effect on pain and structure. However, we just have different opinions. We suppose that anti-TNF-α treatment may be more efficient in patients with -308 allele A genotype because the -308 allele A is associated with about 6 fold increased transcriptional activity and higher protein levels of TNF-α. Therefore, the detection of -308 polymorphism of TNF-α is necessary before the treatment of TNF-α blocker. Further studies are needed to confirm our speculation.

Although the primary results of our meta-analysis are suggestive, some limitations may still exist. Firstly, there was heterogeneity between studies of the -308 polymorphism and the heterogeneity may have distorted the meta-analysis. Unfortunately, meta-regression analysis failed to find the potential heterogeneity. In addition, no quality tool has been used to assess the risk of bias in the included studies, study quality could be one factor missing from the heterogeneity analysis. Secondly, the number of OA cases and studies included in the Caucasian-specific meta-analysis was low. This study may not have enough power to explore the association between the TNF-α -308 polymorphism and risk of OA in the Caucasian population. Thirdly, the results of the meta-analysis were based on unadjusted estimates owing to the lack of adjusted estimates. Currently, some risk factors have been identified, such as aging, obesity, previous injury, smoking habit and hormone therapy. A more precise analysis could be performed if these data were available. Finally, gene-gene interactions were not fully addressed in the meta-analysis because of the lack of relevant data. In fact, the TNF-α gene is located close to the human leucocyte antigen (HLA) class II/III region, in which some SNPs, such as rs7775228 and rs10947262, have been identified as associated with risk of knee OA [30, 34]. Therefore, the positive results from our meta-analysis may result from the linkage disequilibrium of the proximal susceptible SNPs. Further studies on the all SNPs in this region and OA risk are needed.

## Conclusions

In conclusion, this comprehensive meta-analysis has evaluated all published data currently available on the TNF-α -308 polymorphism and risk of OA. Our meta-analysis suggested that the AA and GA genotypes might increase the risk of OA compared with the GG genotype, which may be explained by the higher expression of TNF-α in the -308A allele carriers than in the -308G carriers.

## Electronic supplementary material

Additional file 1: Figure S1: Meta-analysis of TNF-α -308 polymorphism and OA risk after omitting Han’s study: (A) dominant genetic model analysis; (B) recessive genetic model analysis; (C) A allele vs. G allele analysis. (TIFF 12 MB)

Additional file 2: Figure S2: Meta-analysis of TNF-α -308 genotypes and OA risk after omitting Han’s study: (A) AA genotype frequency; (B) GA genotype frequency; (C) GG genotype frequency. (TIFF 12 MB)

Below are the links to the authors’ original submitted files for images.

Authors’ original file for figure 1

Authors’ original file for figure 2

Authors’ original file for figure 3

Authors’ original file for figure 4

Authors’ original file for figure 5

## Abbreviations

TNF-α: Tumor necrosis factor alpha
OA: Osteoarthritis
OR: Odds ratio
CI: Confidence interval
IL-1β: Interleukin 1 beta
HCC: Hospital based case–control study
PCC: Population based case–control study
HWE: Hardy-Weinberg equilibrium
HLA: Human leucocyte antigen.
