# Supplementary figures and images for: Meta-analysis of tumor necrosis factor alpha -308 polymorphism and knee osteoarthritis risk
Source: BMC Musculoskelet Disord. 2014 Nov 15;15:373. doi: 10.1186/1471-2474-15-373 (PMC4289255; doi:10.1186/1471-2474-15-373)

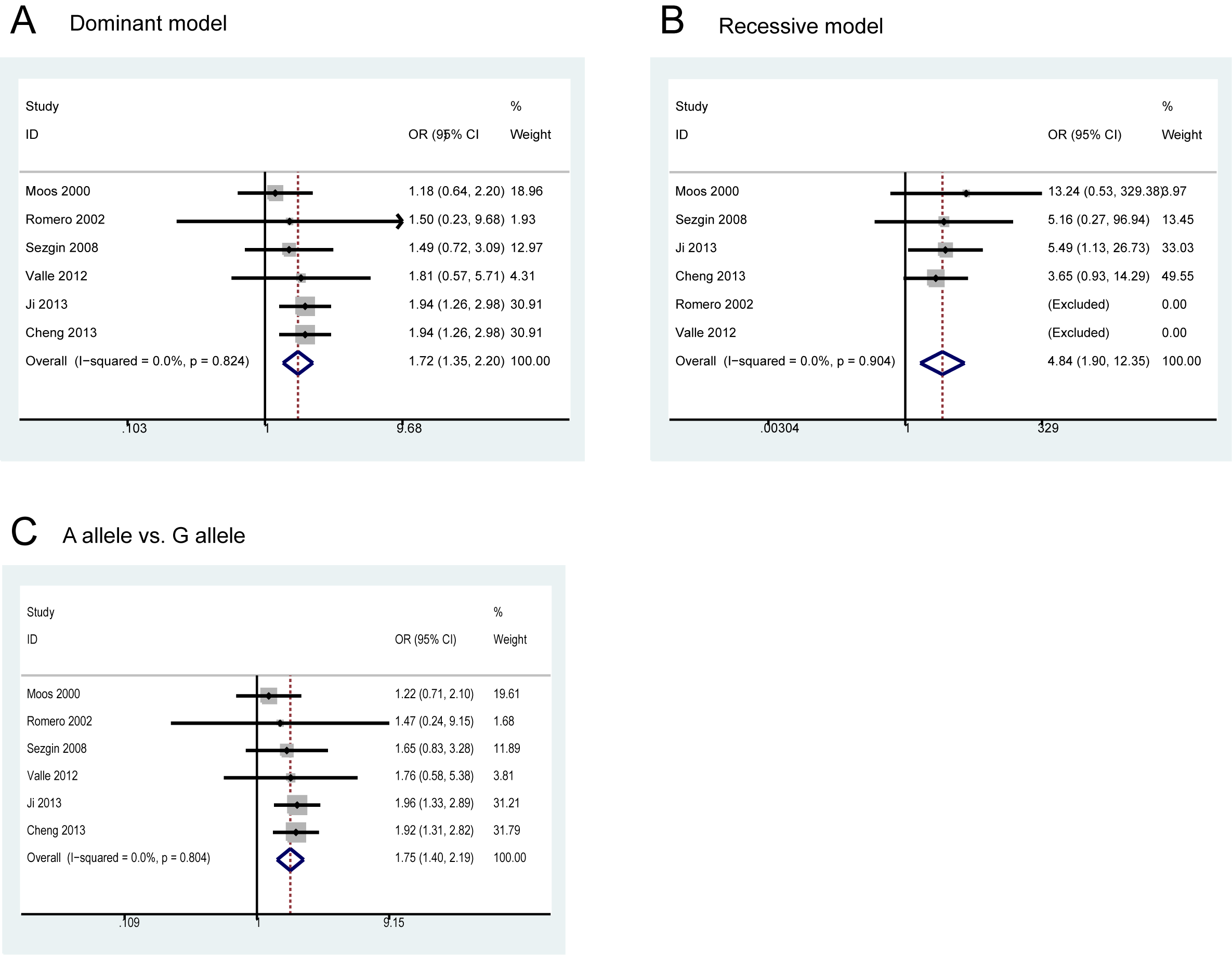

Supplement: Supplementary file 1 — Additional file 1: Figure S1: Meta-analysis of TNF-α -308 polymorphism and OA risk after omitting Han’s study: (A) dominant genetic model analysis; (B) recessive genetic model analysis; (C) A allele vs. G allele analysis. (TIFF 12 MB) [file 12891_2014_2359_MOESM1_ESM.tiff]

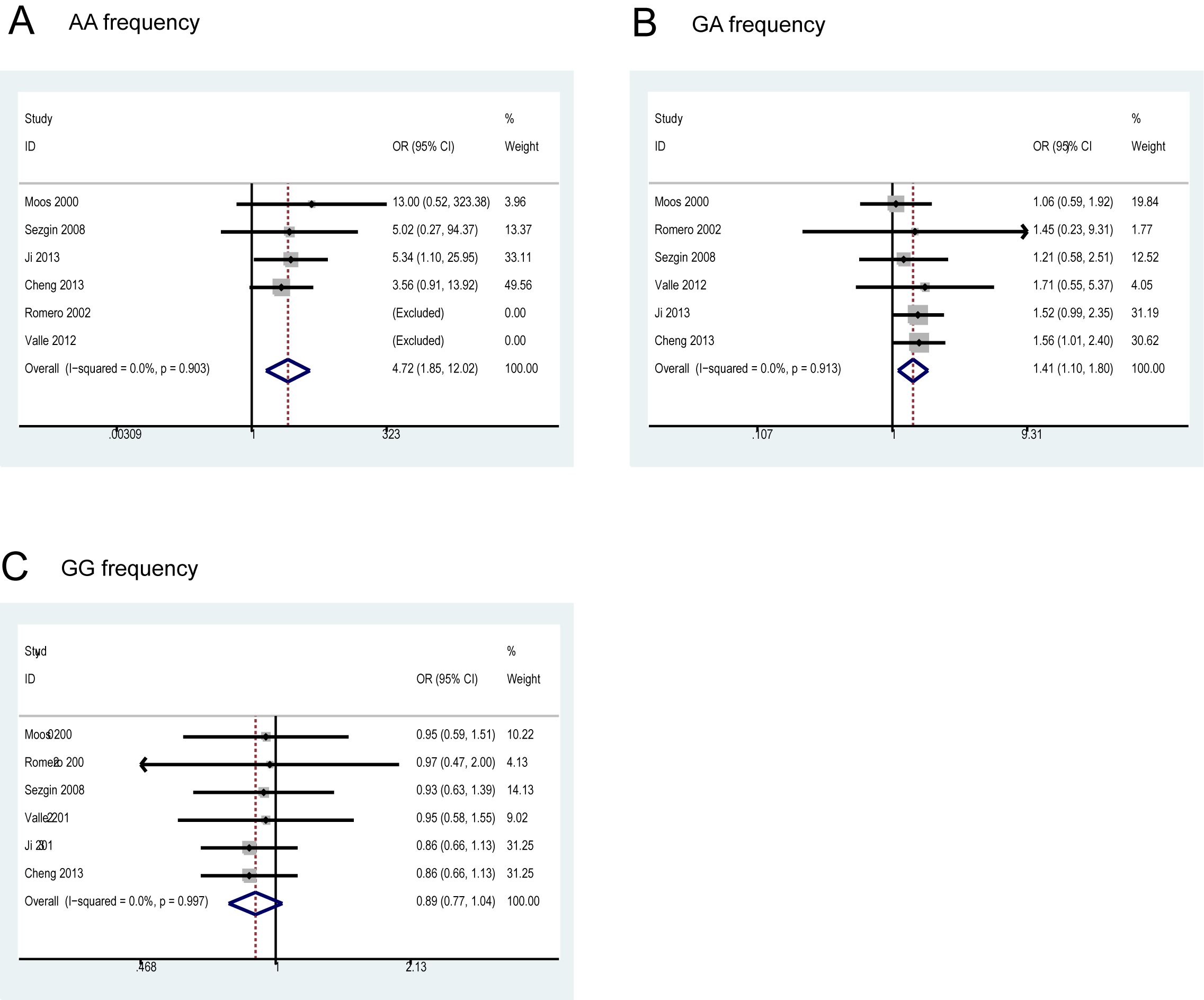

Supplement: Supplementary file 2 — Additional file 2: Figure S2: Meta-analysis of TNF-α -308 genotypes and OA risk after omitting Han’s study: (A) AA genotype frequency; (B) GA genotype frequency; (C) GG genotype frequency. (TIFF 12 MB) [file 12891_2014_2359_MOESM2_ESM.tiff]

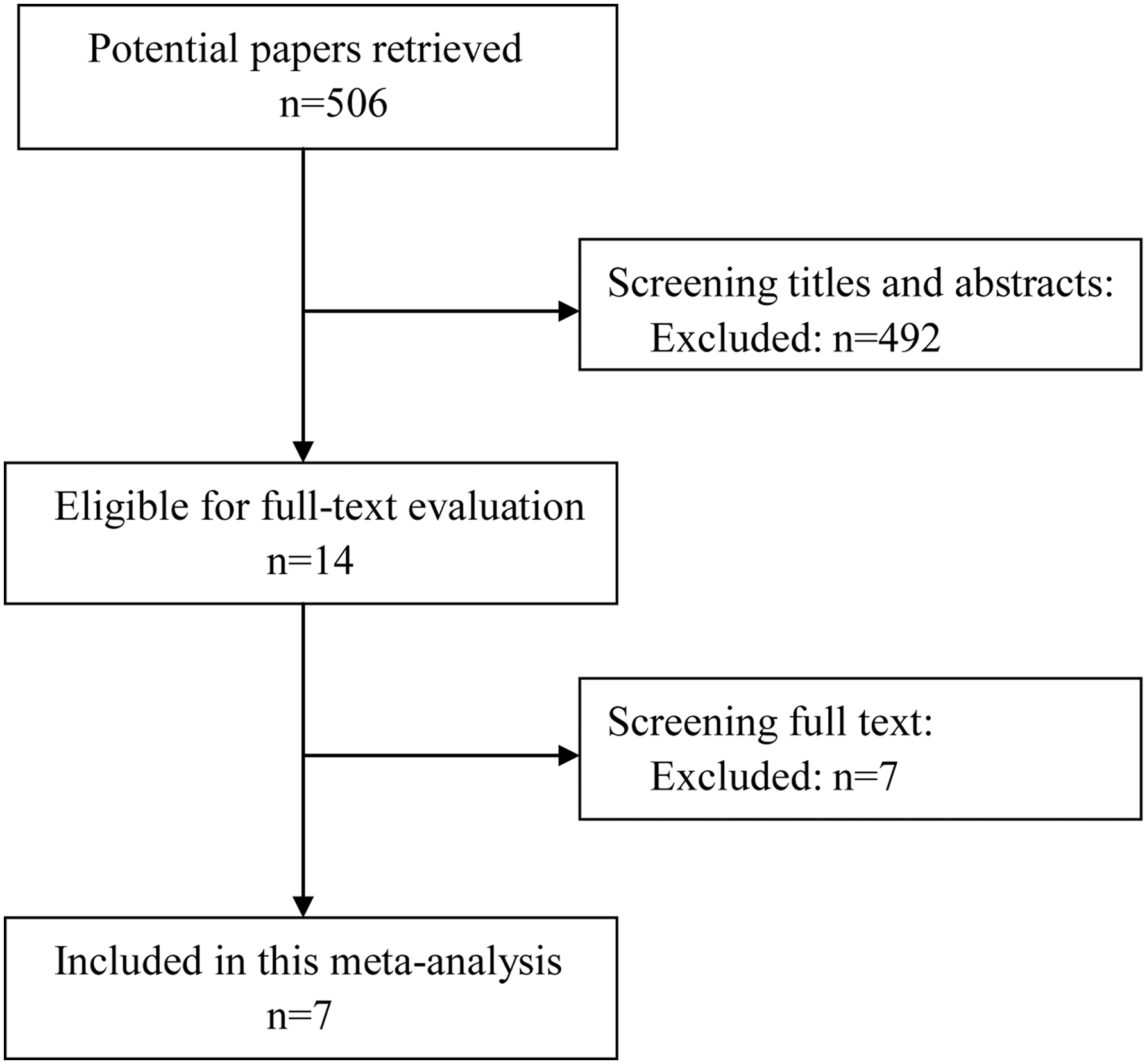

Supplement: Supplementary file 3 — Authors’ original file for figure 1 [file 12891_2014_2359_MOESM3_ESM.tif]

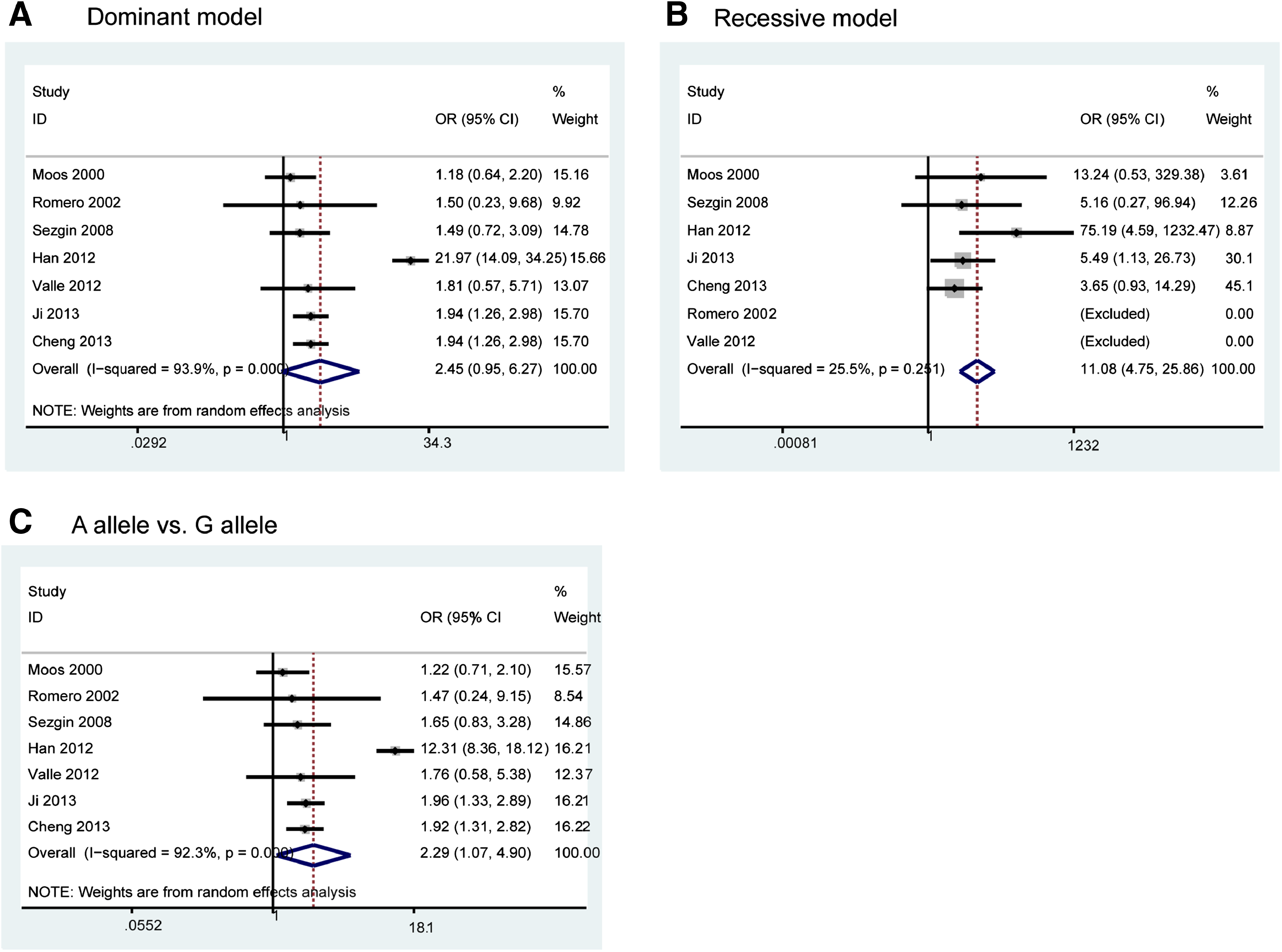

Supplement: Supplementary file 4 — Authors’ original file for figure 2 [file 12891_2014_2359_MOESM4_ESM.tiff]

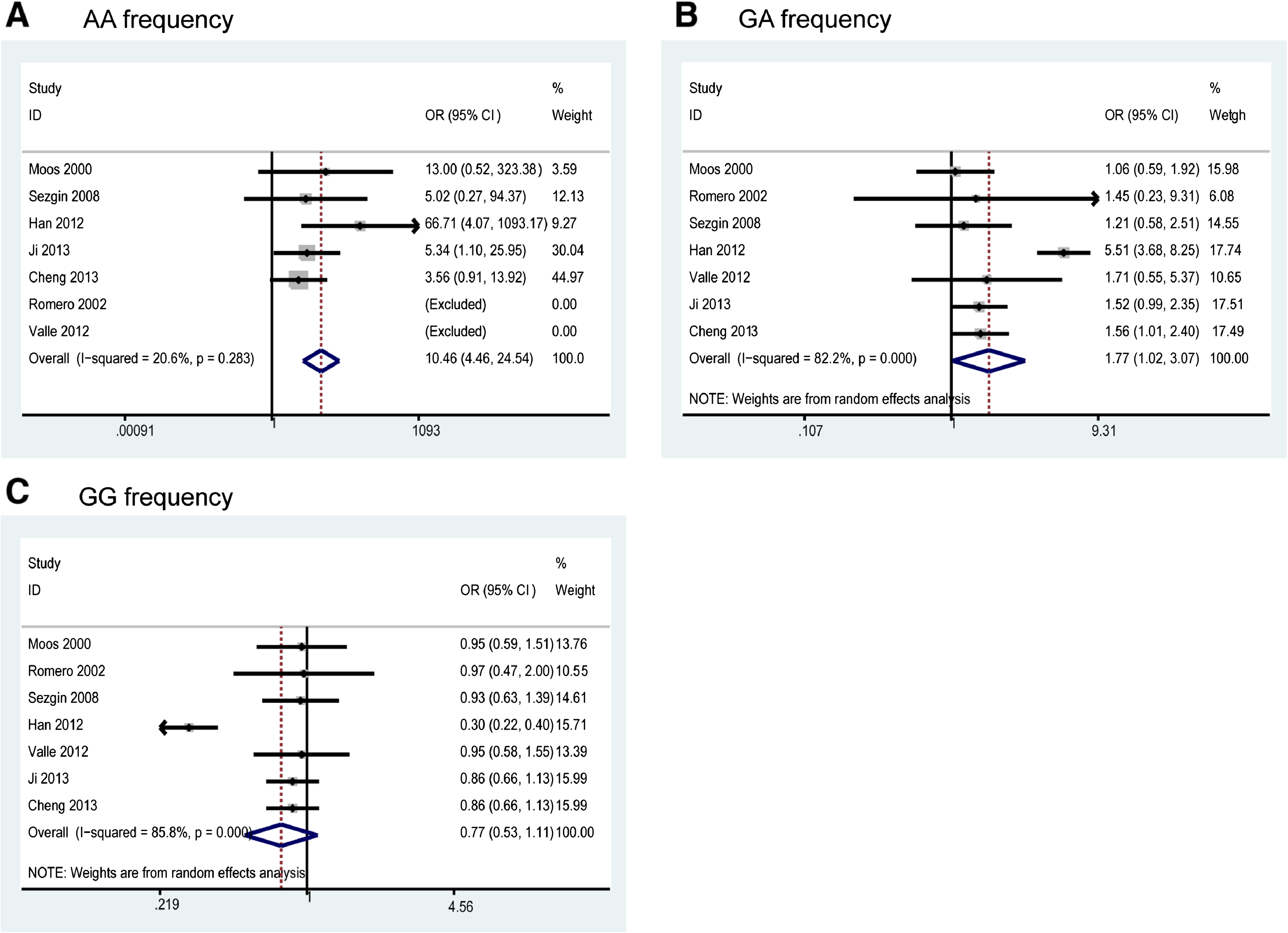

Supplement: Supplementary file 5 — Authors’ original file for figure 3 [file 12891_2014_2359_MOESM5_ESM.tiff]

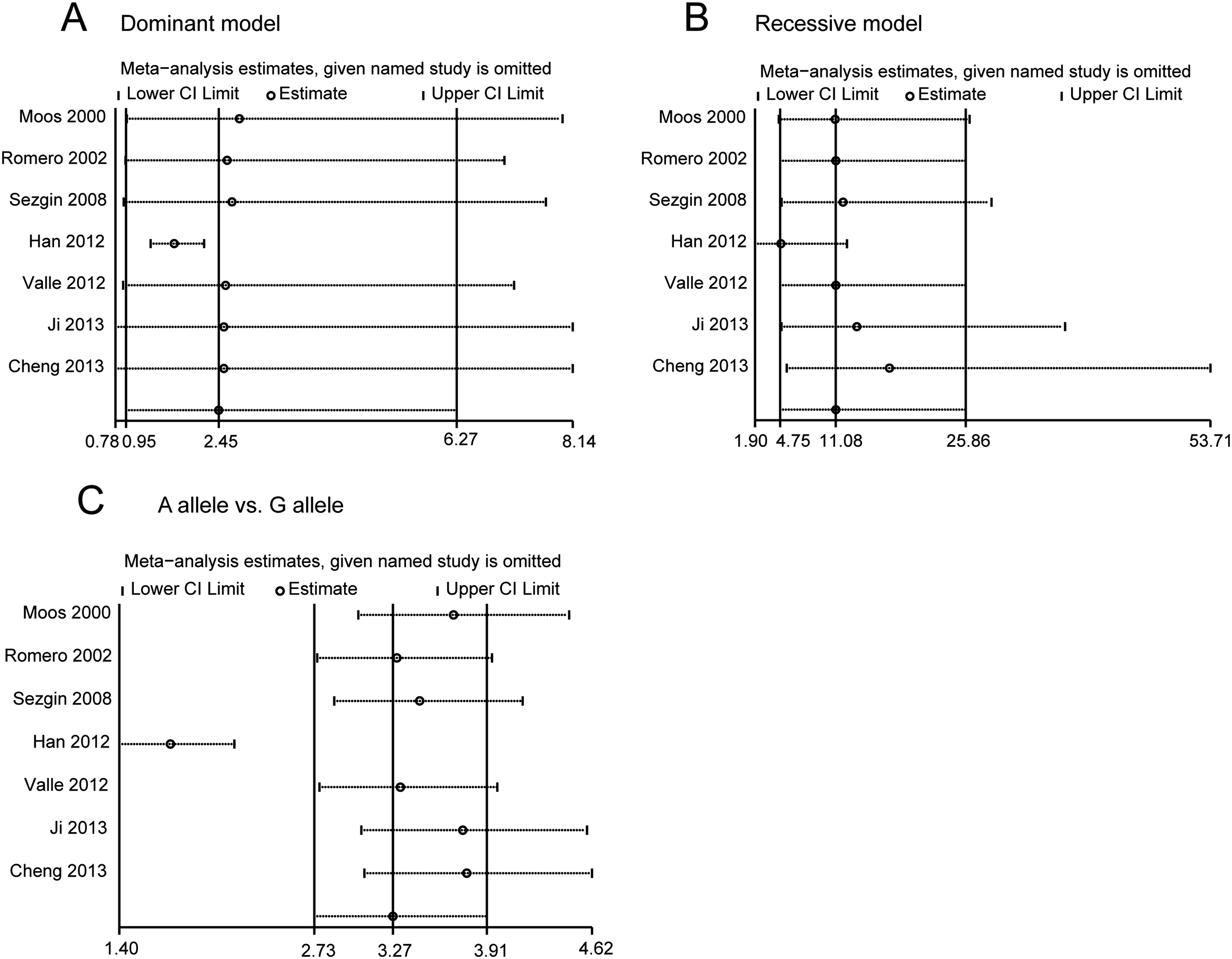

Supplement: Supplementary file 6 — Authors’ original file for figure 4 [file 12891_2014_2359_MOESM6_ESM.tif]

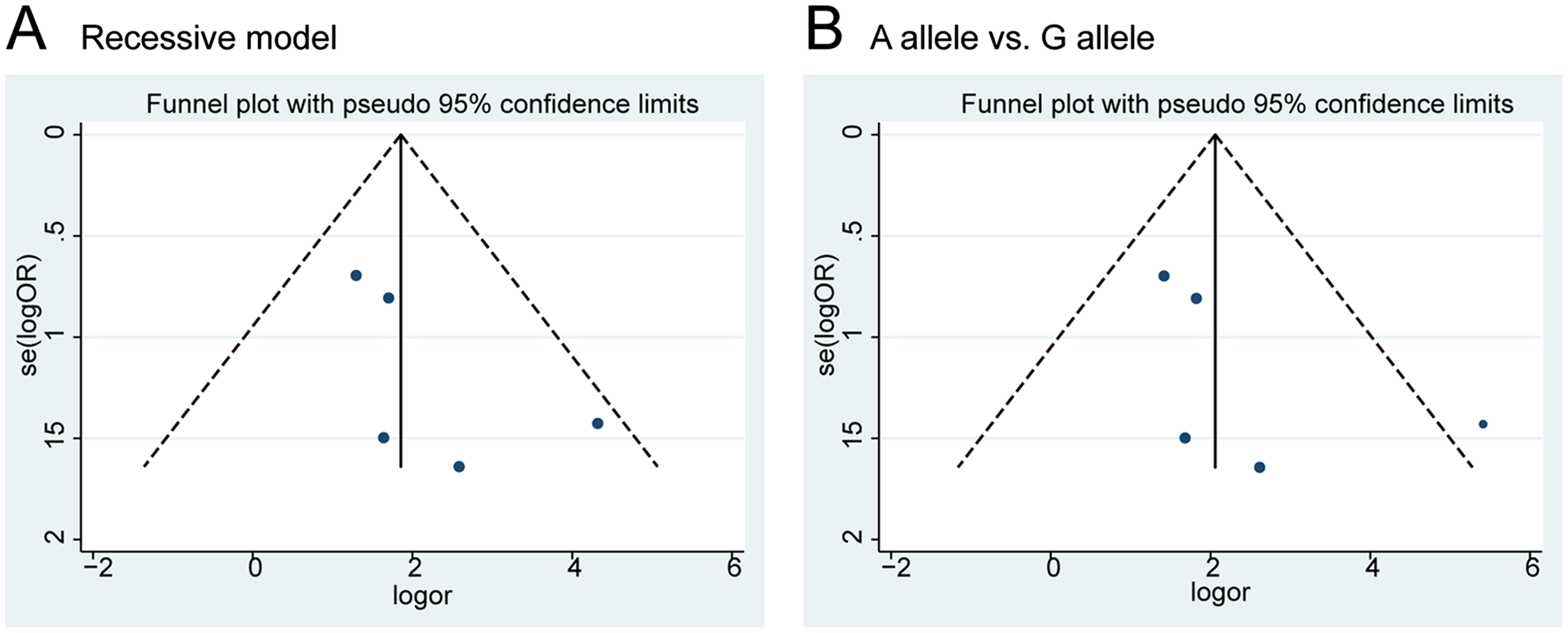

Supplement: Supplementary file 7 — Authors’ original file for figure 5 [file 12891_2014_2359_MOESM7_ESM.tif]
